# Recent Advances in Physiological and Biochemical Responses of Grapevines to Downy Mildew Infection

**DOI:** 10.3390/plants15121917

**Published:** 2026-06-21

**Authors:** Sheng Wang, Tao He, Qi Liu, Mingxin Fu, Naiming Zhang, Li Bao

**Affiliations:** 1 School of Chemistry and Geographical Sciences, Chuxiong Normal University, Chuxiong 675000, China; wangsheng@cxtc.edu.cn; 2 Kunming Institute of Botany, Chinese Academy of Sciences, Kunming 650201, China; hetao1@mail.kib.ac.cn; 3 College of Resources and Environment, Huazhong Agricultural University, Wuhan 430070, China; liuqi67@webmail.hzau.edu.cn; 4 College of Resources and Environment, Yunnan Agricultural University, Kunming 650201, China; 2025210411@stu.ynau.edu.cn

**Keywords:** grapevine, downy mildew, *Plasmopara viticola*, defense signaling network, disease resistance

## Abstract

Grapevine downy mildew, caused by the oomycete pathogen *Plasmopara viticola* (*P. viticola*), is one of the most devastating diseases threatening the global grape industry. The pathogen invades host plants through stomata, triggering a series of highly coordinated physiological disorders and biochemical defense events. This review systematically summarizes the dynamic changes in morphological structures (stomatal characteristics), physiological functions (photosynthesis, membrane system integrity, and carbon metabolism), and multi-level biochemical defense systems (reactive oxygen species (ROS) scavenging enzyme system, phenylpropanoid metabolic pathway, pathogenesis-related proteins, and phenolic compounds) in grapevines following infection. It focuses on analyzing the differences in the timing, intensity, and metabolic reprogramming of defense responses between resistant and susceptible cultivars, pointing out that the essence of disease resistance lies in early pathogen recognition and rapid defense induction. The conflicting conclusions regarding indicators such as soluble sugars, peroxidase (POD), and superoxide dismutase (SOD) are discussed from the perspectives of experimental systems, cultivar genetic backgrounds, and pathogen physiological race differences. Furthermore, the known physiological and biochemical alterations are linked to upstream signaling pathways, including salicylic acid and jasmonic acid (SA/JA), calcium signaling, and mitogen-activated protein kinase (MAPK) cascades. Recent advances in revealing resistance mechanisms in the omics era are also introduced. Finally, future research directions are proposed, including constructing multi-indicator dynamic evaluation models, verifying key gene functions using gene editing, exploring the potential of epigenetic regulation, and developing integrated control strategies combined with microbiome research. This review aims to provide theoretical support for grapevine downy mildew resistance breeding and sustainable disease management.

## 1. Introduction

Grapevine downy mildew, caused by the obligate biotrophic oomycete *P. viticola* (Berk. & M.A. Curtis), is one of the most widely distributed and destructive diseases in viticulture worldwide [1,2,3]. This disease primarily infects young tissues, including leaves, shoots, and immature fruits. The typical symptom is the formation of white downy mildew growth (consisting of sporangiophores and sporangia of the pathogen) on abaxial leaf surfaces, which leads to premature leaf senescence and abscission, shoot necrosis, and fruit shriveling and drop. In severe epidemic years, yield losses can reach up to 80% [2]. The pathogen overwinters as oospores or mycelia in infected plant debris. In the following year, when favorable temperature and humidity conditions occur, sporangia are produced and release zoospores, which invade host plants through stomata to complete the primary and secondary infection cycles [4,5].

For a long time, commercial grape production has mainly relied on chemical fungicides for downy mildew control. However, the intensive and repeated use of fungicides has resulted in the rapid development of fungicide resistance in pathogen populations and reduced control efficacy while also causing serious problems such as pesticide residues and environmental pollution [6]. Therefore, exploiting and utilizing the inherent disease resistance of grapevines has become the core strategy for sustainable disease management. In recent years, extensive research has been conducted on the screening of resistant germplasms and resistance mechanisms at morphological, physiological, biochemical, and molecular levels [7,8]. This review systematically summarizes the physiological and biochemical responses of grapevines to *P. viticola* infection, focusing on membrane integrity, photosynthesis, carbon metabolism, antioxidant enzymes, the phenylpropanoid pathway, PR proteins, and phenolic compounds. It analyzes the differential response patterns between resistant and susceptible cultivars and attempts to link these downstream response events with upstream signaling pathways. This review aims to reveal the physiological and biochemical nature of grapevine resistance to downy mildew and provide a theoretical basis for disease resistance breeding and green disease control.

## 2. Early Infection Stage: The Dual Role of Stomata as a Physical Barrier

Stomata are the primary natural entry route for *P. viticola* zoospores to invade host plants. The relationship between stomatal characteristics and disease resistance remains controversial in existing studies. Some studies have shown that stomatal length, width, and guard cell area are moderately positively correlated with disease resistance, but larger stomatal structures objectively facilitate zoospore attachment, cyst formation, and germ tube penetration [9]. The *P. viticola* can complete infection and produce sporangiophores through stomata [10]. Higher stomatal density and aperture increase the probability of zoospore encounter, thereby enhancing infection success rates.

**Figure 1:**
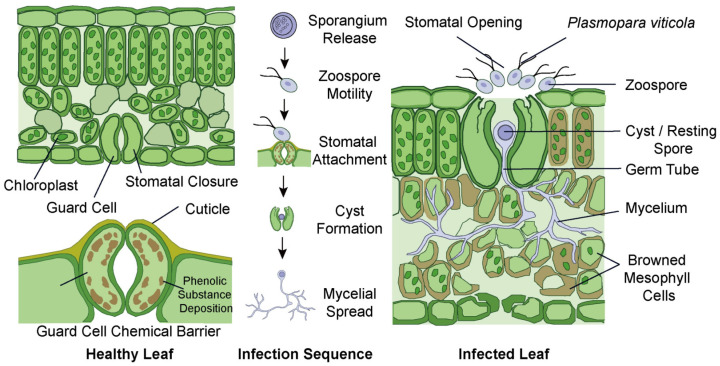
Schematic diagram of structures involved in leaf infection and the infection process of *P. viticola*.

Stomatal characteristics exhibit genetic variations among different grapevine cultivars, but stomatal parameters do not show a simple linear relationship with resistance. Han et al. [11] found that susceptible cultivars had higher leaf stomatal density. However, stomata primarily serve as pathogen entry channels, and disease resistance depends largely on post-invasive recognition and defense activation, including ABA-mediated stomatal immunity. In addition, the chemical composition of guard cells, such as waxes and phenolics, can influence zoospore attachment, while differences in pathogen physiological races contribute to variation in virulence. These factors together may explain the observed discrepancies in the relationship between stomatal density and disease severity [12]. Recent studies have shown that stomata are not only passive channels, but their opening and closing movements are also regulated by immune signals induced by pathogen-associated molecular patterns (PAMPs) such as flg22 and chitin (Figure 1) [13,14]. Plants can actively close stomata as an early “stomatal immunity” strategy to prevent bacterial invasion [15], but whether oomycetes trigger similar responses remains unclear. In the grapevine–*P. viticola* interaction, evidence suggests that pathogen-derived effectors may interfere with stomatal immunity and promote stomatal reopening, thereby facilitating invasion [9,11,16,17]. Therefore, the relationship between stomata and resistance needs to be re-examined in the context of a more complex attack–defense game, and morphological parameters alone are insufficient as resistance indicators.

## 3. Physiological Changes in Grapevines Following Downy Mildew Infection

### 3.1. Plasma Membrane System Damage and Lipid Peroxidation

The plasma membrane is a selective barrier that maintains the stability of the intracellular and extracellular environments. Following *P. viticola* infection, the pathogen causes host cell death by secreting cell wall-degrading enzymes and toxic proteins (effectors) while simultaneously inducing a ROS burst. This triggers a chain reaction of membrane lipid peroxidation, converting the plasma membrane from semipermeable to fully permeable, resulting in massive exudation of intracellular electrolytes and organic molecules [18,19].

Malondialdehyde (MDA) is the main end product of membrane lipid peroxidation, and its content directly reflects the degree of membrane damage. Studies have shown that after grapevine infection with *P. viticola*, cell membranes at sites of hyphal aggregation are dissolved, and both MDA content and relative conductivity increase significantly [20]. Comparisons between cultivars with different resistance levels revealed that MDA accumulation and electrolyte leakage rates were significantly lower in resistant cultivars than in susceptible ones [21]. The cellular mechanisms underlying this difference may include ① resistant cultivars having a lower proportion of unsaturated fatty acids in their membrane lipid composition, making them more tolerant to peroxidation; ② higher contents of membrane-bound antioxidants (α-tocopherol and ubiquinone); and ③ stronger activities of membrane repair-related proteins (phospholipid scramblases and vesicle trafficking-related proteins) [22,23]. In addition, maintenance of plasma membrane integrity depends on intracellular calcium homeostasis. Calcium ion influx is an early signaling event following pathogen infection, but sustained high calcium levels activate calcium-dependent phospholipases, exacerbating membrane degradation [24]. Resistant cultivars may more effectively confine calcium signals within specific spatial and temporal ranges.

### 3.2. Chlorophyll Degradation and Photosynthetic System Damage

A decrease in chlorophyll content is the most intuitive physiological change caused by downy mildew. As disease severity increases, chlorophyll synthesis is inhibited, leading to reduced content and diminished ability of leaves to absorb and utilize light energy [25]. Seven days after inoculation with *P. viticola*, the chlorophyll content of the susceptible cultivar decreased, showing a large and rapid decline. In contrast, resistant genotypes exhibited smaller or insignificant decreases in chlorophyll content [26].

Chlorophyll fluorescence parameters reveal more serious damage to the photosynthetic apparatus. Downy mildew infection significantly inhibits the potential photochemical efficiency of photosystem II (PSII) (Fv/Fm). The magnitude of Fv/Fm reduction in infected areas is positively correlated with disease severity, and this change often precedes visible symptoms [27,28]. This suggests that the pathogen has already inhibited the PSII repair cycle through mechanisms such as toxin secretion, alteration of chloroplast ROS homeostasis, and interference with D1 protein turnover before host cell death [29]. Furthermore, decreased CO_2_ assimilation capacity leads to excess reducing power (NADPH) and adenosine triphosphate (ATP), further exacerbating photoinhibition and ROS production. Resistant cultivars may alleviate photodamage through enhanced non-photochemical quenching (NPQ) and upregulated cyclic electron transport [30,31].

### 3.3. Changes in Gas Exchange Parameters

Following *P. viticola* infection, photosynthetic parameters of grapevines are significantly inhibited, manifested as marked decreases in net photosynthetic rate (*P_n_*) and transpiration rate (*T_r_*), while intercellular CO_2_ concentration (*C_i_*) shows an increasing trend [26]. Further studies by Júnior et al. [32] in grapevine cultivars with different resistance levels demonstrated that as disease severity increased, *P_n_* and *T_r_* in resistant cultivars decreased but to a lesser extent, while susceptible cultivars exhibited more pronounced photosynthetic inhibition, including decreased maximum carboxylation rate of Rubisco, reduced photochemical efficiency, and significantly elevated *C_i_*. Similar results have been reported in the cucumber downy mildew system, indicating that downy mildew infection generally leads to reduced photosynthetic efficiency and structural damage to the photosynthetic system in hosts [33].

The characteristic pattern of decreased *P_n_* accompanied by increased *C_i_* indicates that photosynthetic limitation primarily arises from non-stomatal factors, i.e., damage to the photosynthetic apparatus within mesophyll cells, rather than stomatal closure. The underlying mechanisms may involve oxidative inactivation of key enzymes such as Rubisco caused by ROS accumulation, inhibition of Calvin cycle key enzymes (glyceraldehyde-3-phosphate dehydrogenase), and dysfunction of the photosynthetic electron transport chain due to damage to chloroplast membrane systems [34]. In addition, the reduction in *T_r_* not only reflects decreased stomatal conductance but may also be related to impaired mesophyll conductance, a process closely associated with downregulated expression or activity of plasma membrane intrinsic proteins (PIPs) [35].

### 3.4. Soluble Sugar Content: Temporal Dynamics Behind the Contradictions

Soluble sugars are not only important carbon sources and energy reserves in plants but also crucial metabolic nodes for osmotic regulation, stress signal transduction, and defense response regulation [36]. Existing studies have shown that changes in soluble sugar content following pathogen infection do not follow a single trend but are jointly influenced by multiple factors, including host genotype, pathogen type, infection stage, and environmental stress background. Mahatma et al. [37] found significant differences in leaf metabolite composition between resistant and susceptible pearl millet genotypes to downy mildew, suggesting that carbohydrate metabolism may be involved in host defense responses. Xin et al. [38] pointed out in rice blast research that soluble sugar and soluble protein contents have certain correlations with seedling disease resistance, indicating that soluble sugars can serve as potential physiological and biochemical indicators for evaluating plant disease resistance. Similarly, studies on cucumber downy mildew have demonstrated that alterations in photosynthesis and carbohydrate metabolism accompany disease development, further supporting the use of carbohydrate-related metabolites as indicators of plant responses to downy mildew stress [39].

However, the relationship between soluble sugar content and resistance is not entirely consistent across different studies: some studies suggest that resistant materials can maintain higher or more stable soluble sugar levels in the early infection stage, while others find that susceptible materials show more pronounced sugar accumulation during disease development [40,41]. Therefore, the relationship between soluble sugars and downy mildew resistance is not simply positive or negative but rather reflects a dynamic metabolic response process that changes with infection progression. These differences can be explained by the temporal dynamics of carbon metabolism and the remodeling of "source–sink" relationships during pathogen infection. In the very early stage of infection, resistant materials may rapidly recognize the pathogen and regulate sugar transport and signaling pathways, promoting the transient accumulation of soluble sugars to serve as energy substrates, osmotic regulators, and signaling molecules for defense gene activation. In the middle stage of infection, soluble sugars may be further redirected to defense-related processes such as respiratory metabolism, phenolic compound synthesis, and lignin deposition, thus showing a decline in content or maintenance of relative stability [42].

In contrast, susceptible materials exhibit delayed defense activation, which promotes pathogen spread and cellular damage, reduces membrane stability, impairs photosynthate export and source–sink balance, and ultimately leads to the continuous accumulation or irregular fluctuation of soluble sugars [43,44]. Studies on grapevine downy mildew have also shown that pathogen infection can significantly affect leaf photosynthesis and defense-related transcriptional processes, providing physiological and molecular evidence for the dynamic changes in soluble sugar content with infection progression [26,45].

Therefore, the absolute content of soluble sugars alone is insufficient to determine material resistance. Its early accumulation rate, mid-stage consumption or conversion rate, and late fluctuation pattern may better reflect the true state of the host’s defense response. Future research should focus on the dynamic regulation of soluble sugar metabolism during pathogen infection by continuously monitoring soluble sugar accumulation at key infection stages (0–72 h post-inoculation). Combined analyses of sugar metabolism-related enzymes, hexokinase-dependent sugar signaling, and downstream defense-responsive genes would help clarify how carbohydrate metabolism and sugar signaling coordinate host defense responses, thereby contributing to the development of resistance against downy mildew [46,47].

## 4. Activation of Biochemical Defense Systems in Grapevines Following Downy Mildew Infection

**Figure 2:**
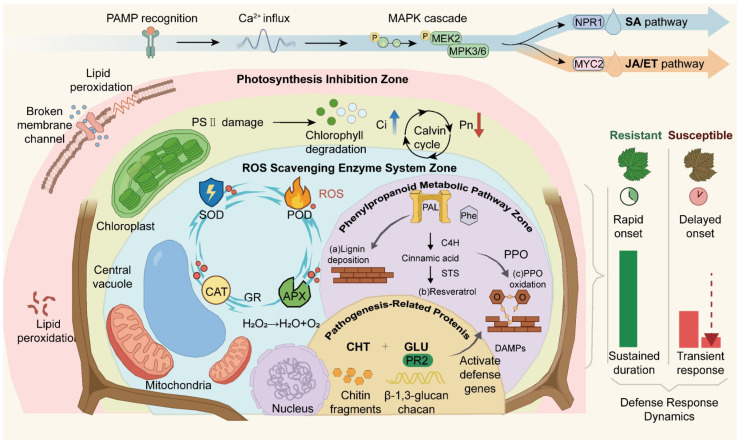
Multi-level defense mechanisms of grapevines in response to downy mildew pathogen infection.

Upon infection by *P. viticola*, grapevines activate a multi-level biochemical defense network, including ROS scavenging enzyme systems, the phenylpropanoid metabolic pathway, PR proteins, and phenolic compound accumulation (Figure 2) [48].

### 4.1. Reactive Oxygen Species Scavenging Enzyme System

#### 4.1.1. Superoxide Dismutase (SOD)

The SOD is a key enzyme in the plant ROS scavenging system, which catalyzes the dismutation of superoxide anions (O^2−^) into H_2_O_2_ and O_2_, thereby limiting excessive O^2−^ accumulation and maintaining cellular redox homeostasis [49]. Changes in SOD activity following downy mildew infection are generally closely related to host resistance. Studies have shown that exogenous melatonin can enhance antioxidant enzyme activities and improve downy mildew resistance in cucumbers [50]; kaolin particle film treatment can also enhance grapevine resistance to downy mildew by inducing defense responses, with regulation of ROS metabolism being an important link [48]. These results indicate that a timely and effective SOD response helps scavenge excess O^2−^, reduce oxidative damage, and maintain defense signal transduction. However, together with evidence that resistant and susceptible grapevine cultivars activate distinct defense strategies, these findings suggest that the relationship between SOD activity and disease resistance is complex and cannot be interpreted as simply positive [51]. This discrepancy may arise from the dual role of ROS in plant–pathogen interactions. Moderate ROS accumulation can act as signaling molecules to participate in defense response activation, while excessive ROS leads to membrane lipid peroxidation, protein inactivation, and cellular structural damage [52]. Therefore, the role of SOD in disease resistance depends not only on its activity level but also on its induction timing, response intensity, and coordination with downstream H_2_O_2_ scavenging systems. Resistant materials usually rapidly induce SOD activity in the early infection stage, converting O^2−^ to H_2_O_2_, which has dual signaling functions, thereby promoting defense response initiation while controlling oxidative damage. In contrast, higher SOD activity in susceptible materials may be more indicative of passive stress responses induced by ROS accumulation after pathogen spread; if their response is delayed or fails to coordinate with peroxidase (POD), catalase (CAT), and the ascorbate–glutathione cycle, it may still lead to H_2_O_2_ accumulation and exacerbate oxidative damage [53,54]. Therefore, the induction timing and peak intensity of SOD are more important than its final activity level.

#### 4.1.2. Peroxidase (POD)

The POD participates in plant cell wall reinforcement, ROS homeostasis maintenance, and phenolic compound oxidation, making it an important defense-related enzyme following pathogen infection. On the one hand, POD promotes lignin polymerization to strengthen the physical barrier of cell walls; on the other hand, its mediated phenolic oxidation products may directly inhibit pathogen spread [48]. Existing studies have reported inconsistent relationships between POD activity and resistance: some studies showed that susceptible cultivars exhibited greater increases in POD activity after downy mildew infection [55], while others found that resistant materials had higher POD activity levels or stronger induction amplitudes [56,57]. This discrepancy may be related to the POD isozyme composition and functional differentiation. Different POD isozymes differ in subcellular localization, substrate preference, and physiological functions. Cell wall-bound acidic POD is more likely to be involved in lignification and structural defense, while some cytosolic or alkaline PODs mainly participate in H_2_O_2_ scavenging and redox regulation [58]. These findings suggest that the contribution of POD to disease resistance depends not only on its overall activity but also on the specific functions of individual POD isoforms and their coordination within the antioxidant defense network [59,60]. Thus, simply comparing total POD activity is difficult to accurately explain its role in defense responses. Distinguishing POD isozyme types through native PAGE, isozyme profile analysis, and substrate specificity determination is more meaningful than total POD activity.

#### 4.1.3. Catalase (CAT)

The CAT is an important antioxidant enzyme in plants that decomposes H_2_O_2_ into H_2_O and O_2_, primarily located in peroxisomes. Studies have shown that CAT activity is usually significantly increased in resistant materials after downy mildew infection, while it remains low or decreases in the late infection stage in susceptible materials [45,61]. However, some studies have also reported a downward trend in CAT activity during induced defense processes [48]. Its role may depend on H_2_O_2_ accumulation levels, infection periods, and the synergistic status of other antioxidant enzyme systems.

Compared with ascorbate peroxidase (APX) and some peroxidases, CAT has a lower affinity for H_2_O_2_ and usually plays a major scavenging role when H_2_O_2_ concentrations are high [62]. In the early stage of pathogen infection, resistant cultivars may preferentially use higher-affinity APX and peroxidases (PRX) to finely regulate H_2_O_2_ signals, while in the late infection stage, when H_2_O_2_ is produced in large quantities, CAT is activated to prevent oxidative damage. Susceptible cultivars may experience CAT release or inactivation due to impaired peroxisome integrity. Therefore, the change pattern of the CAT/APX ratio can serve as a resistance indicator: resistant cultivars show APX dominance in the early stage and CAT elevation in the late stage, while susceptible cultivars maintain persistently low CAT levels, and APX is also prone to inactivation.

#### 4.1.4. Ascorbate–Glutathione Cycle and APX

The APX catalyzes H_2_O_2_ reduction using ascorbate (AsA) as an electron donor and is an important enzyme for finely regulating intracellular H_2_O_2_ levels in plant cells [63]. Ascorbate peroxidase (APX), together with monodehydroascorbate reductase (MDHAR), dehydroascorbate reductase (DHAR), and glutathione reductase (GR), constitutes the ascorbate–glutathione (AsA–GSH) cycle. By continuously regenerating ascorbate (AsA) and glutathione (GSH), this cycle maintains cellular redox balance and represents one of the most important H_2_O_2_-scavenging pathways in ROS-producing organelles, especially chloroplasts [63,64,65]. Transgenic studies have further demonstrated the critical role of APX in oxidative stress defense. Previous studies have shown that chloroplast-targeted APX overexpression enhances antioxidant capacity and protects the photosynthetic machinery by maintaining higher photosynthetic rates and PSII photochemical efficiency under oxidative stress [66]. Nazish et al. [61] demonstrated that overexpression of thylakoid APX in Arabidopsis thaliana mitigates paraquat-induced photooxidative stress and enhances cellular ROS scavenging [67]. These results indicate that chloroplast APX and its associated antioxidant network play important roles in maintaining photosynthetic system stability and limiting ROS damage. In the grapevine–*P. viticola* interaction, studies have found that the reduced state ratios of AsA and GSH pools (AsA/DHA, GSH/GSSG) in leaves of resistant cultivars are significantly higher than those of susceptible cultivars, even when total antioxidant capacities are similar. This suggests that the key to resistance in resistant cultivars lies in higher activities of regeneration systems (MDHAR, DHAR, and GR), which allow rapid reduction and regeneration of oxidants [68].

### 4.2. Key Enzymes of the Phenylpropanoid Metabolic Pathway

#### 4.2.1. Phenylalanine Ammonia-Lyase (PAL)

The PAL is the key rate-limiting enzyme of the phenylpropanoid metabolic pathway, which catalyzes the deamination of phenylalanine to produce cinnamic acid, thereby initiating the synthesis of various defense-related metabolites, including lignin, phenolic acids, flavonoids, and stilbenes [69]. This pathway plays a central role in grapevine defense responses against downy mildew. Its products can not only strengthen cell wall structural barriers through lignin deposition but also directly inhibit pathogen spread via antimicrobial substances such as chlorogenic acid, flavonoids, and resveratrol [70]. Recent physiological and transcriptomic studies have shown that the accumulation of phenylpropanoid metabolites in grapevine leaves is closely correlated with downy mildew resistance [21]; exogenous thiamine-induced grapevine resistance to downy mildew is also accompanied by significant activation of the phenylpropanoid metabolic pathway [71]. In terms of response patterns, resistant cultivars exhibit a large induction amplitude of PAL activity, with an early and long-lasting activity peak [72]. The five PAL family members (*VvPAL1-5*) show distinct expression patterns in grapevines. Studies have indicated that several PAL family members, including VvPAL1, VvPAL2, and VvPAL5, have been reported to be induced following *P. viticola* infection, although their expression patterns may vary among cultivars and infection stages [73].

Phenylpropanoid metabolites also exhibit clear spatial division of labor at the tissue and subcellular levels: lignin is mainly deposited in cell walls and vascular tissues around infection sites, promoting papilla formation and cell wall reinforcement; phenolic substances such as chlorogenic acid are mostly accumulated in vacuoles and can be released to participate in local antimicrobial reactions when pathogens invade or cells are damaged. This spatial compartmentalization and coordination are important features of disease resistance mechanisms [74,75].

#### 4.2.2. Polyphenol Oxidase (PPO)

The PPO is a key enzyme in plant phenolic oxidative metabolism, which mainly catalyzes the oxidation of phenolic substrates to produce quinone compounds [76]. Quinones have strong antimicrobial activity, can covalently modify pathogen proteins and enzymes, and simultaneously promote cell wall lignification [77]. In line with these findings, PPO activity was rapidly and strongly induced in the resistant genotype Garovilli following *Macrophomina phaseolina* infection, whereas it remained low and declined in the susceptible genotype H 174 [78]. For a long time, PPO has been considered to be stored in chloroplasts or plastids in the form of inactive precursors (latent pro-PPO), strictly spatially separated from phenolic substrates stored in vacuoles. Membrane system disturbances, Ca^2+^ influx, or cellular structural damage caused by pathogen infection can promote the contact of PPO with substrates such as chlorogenic acid and catechol, triggering phenolic oxidation reactions and generating quinone products with antimicrobial and antinutritional properties, thereby limiting pathogen spread [79,80]. Resistant cultivars can more effectively maintain PPO mRNA stability and induce the synthesis of precursor proteins, while susceptible cultivars may experience PPO degradation due to excessive protease activity.

### 4.3. Pathogenesis-Related Proteins (CHT and GLU)

Chitinase (CHT; PR-3, PR-4, PR-8, and PR-11) and *β*-1,3-glucanase (GLU; PR-2) are important members of plant pathogenesis-related proteins. They are often induced after pathogen infection and participate in processes such as cell wall degradation, antimicrobial substance release, and defense signal amplification [81]. These two enzymes can synergistically hydrolyze structural polysaccharides in pathogen cell walls, thereby inhibiting pathogen spread. Although *P. viticola* is an oomycete whose cell walls are mainly composed of *β*-1,3-glucan and cellulose with low or no chitin content, GLU can still directly act on its cell wall components; CHT may indirectly enhance host defense responses by recognizing or hydrolyzing structurally similar glycosidic bonds or promoting the release of oligosaccharide elicitors [82].

Studies have confirmed that CHT and GLU activities and gene expression are significantly increased in resistant cultivars after inoculation with *P. viticola* [51,83,84], and their antifungal activity is greatly enhanced when acting synergistically [85]. Numerous studies have found that there are two types of GLU in grapevine leaves: alkaline GLU is localized in vacuoles, while acidic GLU is secreted into the intercellular spaces. Since the early stage of *P. viticola* infection mainly occurs in intercellular spaces and mesophyll tissues, secreted GLU can contact pathogen structures earlier and participate in local defense responses. Endo-*β*-1,3-glucanase with anti-*P. viticola* activity has been identified in grapevines, indicating that GLU can directly participate in the inhibition of downy mildew [86].

Meanwhile, PR proteins not only have direct antimicrobial activity but also interact with the plant immune signaling network, playing extensive regulatory functions in responses to both biotic and abiotic stresses [87]. Furthermore, the hydrolysis products of CHT and GLU (chitooligosaccharides and glucans) can act as damage-associated molecular patterns (DAMPs) to further amplify defense signals, forming a positive feedback loop [88].

### 4.4. Phenolic Compounds: From End Products to Signaling Hubs

Phenolic compounds are the end products of phenylpropanoid metabolism, including phenolic acids (chlorogenic acid, ferulic acid), flavonoids (catechin, proanthocyanidins), and stilbenes (resveratrol). They can inhibit pathogen cell wall-degrading enzymes, directly inactivate pathogens, enhance cell wall mechanical strength, and act as signaling molecules to activate downstream defense responses [89]. Studies have shown that the contents of chlorogenic acid, ferulic acid, proanthocyanidins, catechin, and total phenolics in fruits or leaves of resistant cultivars exhibited significantly greater increases after inoculation than those of susceptible cultivars [90,91]. Resveratrol, a phytoalexin unique to grapevines, is rapidly synthesized and accumulated after *P. viticola* infection, and its content is highly correlated with disease resistance. Stilbene synthase (STS), the key enzyme catalyzing the condensation of one molecule of coumaroyl-CoA with three molecules of malonyl-CoA to form resveratrol, belongs to the type III polyketide synthase (PKS) family [92]. Unlike other stilbene-producing plants, the grapevine genome (cv. PN40024) contains an exceptionally large STS multigene family, whose large-scale expansion itself constitutes the genetic basis for grapevine disease resistance potential [90,93,94]. Pathogen-induced STS expression varies among cultivars, with resistant cultivars generally exhibiting stronger and more rapid induction of STS genes following pathogen infection [95]. Furthermore, phenolic compounds can be oxidized by PPO to quinones, which can cross-link cell wall proteins to form an impermeable barrier. Covalent linkages between phenolics and cell wall polysaccharides (ferulic acid bridging of arabinoxylans) are also important mechanisms for enhancing cell wall resistance to degradation.

### 4.5. Upstream Signaling Pathways: From Physiological and Biochemical Responses to Molecular Regulation

The physiological and biochemical events described above do not occur in isolation but are controlled by a highly regulated signaling network. Pathogen-associated molecular patterns (conserved microbial molecules derived from *P. viticola*, including β-glucans and other pathogen-associated molecular patterns (PAMPs)) are perceived by plant pattern-recognition receptors (PRRs) on the plant plasma membrane, triggering PAMP-triggered immunity (PTI) [96]. This process involves ① activation of plasma membrane NADPH oxidase (RBOH) leading to ROS burst; ② calcium ion influx activating calcium-dependent protein kinases (CDPKs) and calmodulins; and ③ phosphorylation of transcription factors by MAPK cascades (MEK2-MPK3/MPK6) [97,98]. These signals ultimately lead to the expression of downstream defense genes (PAL, STS, CHT, and GLU). SA and JA/ethylene (ET) are the major endogenous hormonal signals. It is generally accepted that the SA pathway plays a dominant role against biotrophic pathogens (downy mildew), while the JA/ET pathway is effective against necrotrophic pathogens. In the grapevine–*P. viticola* interaction, resistant cultivars exhibit earlier and higher SA accumulation, which triggers systemic acquired resistance (SAR), whereas susceptible cultivars may experience abnormal activation of the JA pathway or dysregulation of the SA/JA balance. Although existing studies have verified the roles of these signals through exogenous SA or methyl jasmonate treatments, the fine-tuned crosstalk between SA and JA in grapevine downy mildew still requires in-depth exploration [99].

## 5. Conclusions and Perspectives

### 5.1. Conclusions

**Figure 3:**
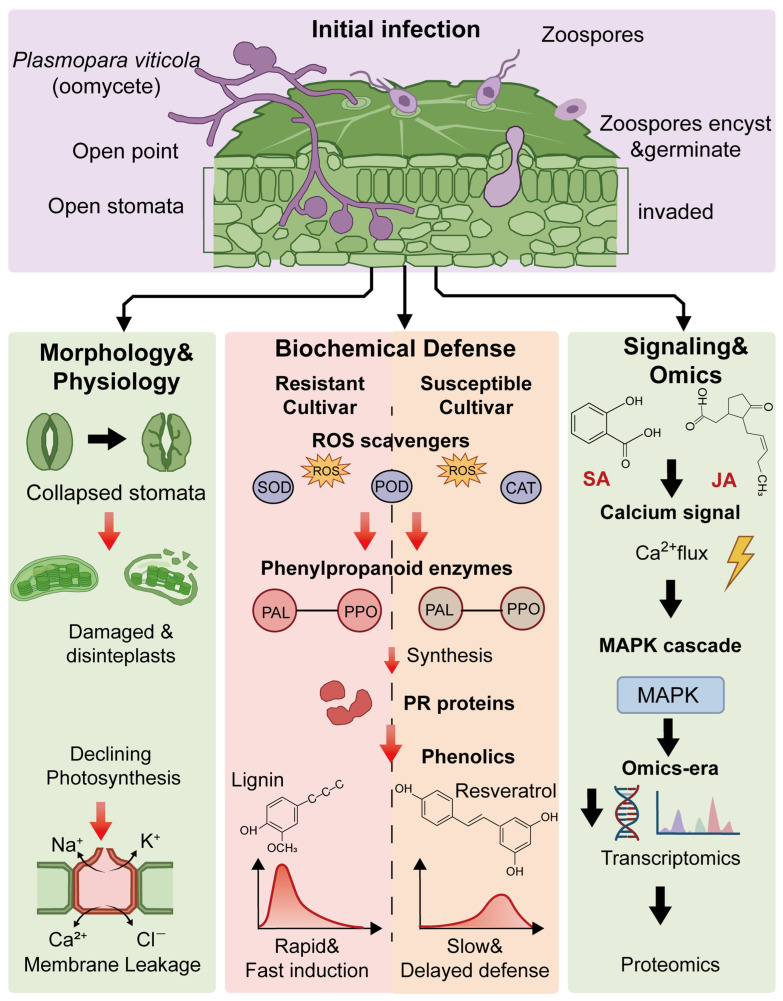
Multi-level defense response model of grapevines under *P. viticola* infection.

This review systematically summarizes the research progress on the physiological and biochemical responses of grapevines to downy mildew infection. At the physiological level, pathogen infection leads to intensified membrane lipid peroxidation (MDA↑, electrical conductivity↑), impaired photosynthesis (Chl↓, Fv/Fm↓, *P_n_*↓, *C_i_*↑), and carbon metabolism reprogramming (dynamic fluctuations of soluble sugars). Resistant cultivars exhibit milder damage and faster recovery capacity in these indicators. At the biochemical level, grapevines activate a multi-level defense system, including ROS scavenging enzyme systems (SOD, POD, CAT, and APX), phenylpropanoid metabolic enzymes (PAL, PPO), pathogenesis-related proteins (CHT, GLU), and phenolic compound accumulation. The core characteristics distinguishing resistant cultivars from susceptible ones lie in the timeliness, intensity, and sustainability of defense responses, rather than the absolute magnitude of any single indicator. Furthermore, the roles of physical structures such as stomata cannot be considered in isolation but need to be comprehensively evaluated in combination with chemical barriers and induced defenses (Figure 3, Table 1 and Table 2).

### 5.2. Research Perspectives

Although existing studies have outlined the physiological and biochemical landscape of grapevine–*P. viticola* interactions, there remains considerable room for improvement in mechanistic depth, integration level, and translational application:

(1) Constructing multi-indicator dynamic evaluation models: Current resistance identification mostly relies on field disease indices or enzyme activity measurements at single time points, which lack sufficient information dimensions. It is recommended that future studies utilize high-temporal-resolution phenotyping platforms combined with technologies such as chlorophyll fluorescence imaging, thermal imaging, and multispectral reflectance to simultaneously acquire high-frequency data on photosynthesis, transpiration, and antioxidant status. Machine learning algorithms (random forests, support vector machines) can then be employed to establish resistance prediction models based on early physiological and biochemical characteristics. This will provide non-destructive and high-throughput tools for large-scale germplasm screening.

(2) Moving from correlation analysis to causal relationship verification: Numerous studies have confirmed correlations between certain enzyme activities and resistance, but functional validation is lacking. Technologies such as virus-induced gene silencing (VIGS), CRISPR/Cas9, or overexpression should be used to functionally validate candidate genes such as *VvPAL1*, *VvSTS*, and *VvGLU* in the grapevine endogenous system, clarifying their necessity and sufficiency in disease resistance pathways. Meanwhile, heterologous expression systems (transient expression in tobacco) combined with effector co-infiltration can enable high-throughput screening of plant targets for pathogen effector proteins.

(3) Integrating omics to reveal regulatory networks: Single-indicator studies can no longer meet the demands of the systems biology era. It is suggested to conduct a joint analysis of time-series transcriptomics (RNA-seq) and metabolomics (LC-MS/MS) to construct gene–metabolite co-expression networks and identify core regulatory nodes (transcription factors such as WRKY, MYB, and NAC). Metabolic flux analysis (^13^C labeling) can reveal the dynamic allocation of carbon and nitrogen between primary and secondary metabolism. Furthermore, single-cell transcriptomics technology holds promise for resolving the heterogeneous responses of different cell types (guard cells, mesophyll cells, vascular cells) around pathogen infection sites.

(4) Focusing on epigenetic regulation and environmental adaptability: The occurrence of downy mildew is highly dependent on temperature and humidity. Investigating the roles of DNA methylation and histone modifications (H3K4me3, H3K9ac) in resistance induction and memory may reveal epigenetic variation-mediated resistance priming phenomena. Meanwhile, integrating multi-omics analysis of adaptive differences to downy mildew among different grapevine ecotypes will provide a basis for breeding broadly adaptable cultivars.

(5) Exploring the potential of the microbiome and rhizosphere immunity: Grapevine leaves and rhizospheres harbor complex microbial communities, and certain beneficial bacteria can enhance grapevine resistance to downy mildew through induced systemic resistance (ISR). Future research can use physiological and biochemical indicators as tools for evaluating the efficacy of microbial agents and should explore microbiome transplantation to restore resistance.

(6) Promoting the efficient utilization of resistance resources: Chinese wild grapevine resources (*Vitis pseudoreticulata*, *Vitis amurensis*) retain abundant resistance genes. It is recommended to systematically evaluate the physiological and biochemical characteristics of these resources (such as PAL induction rate, MDA accumulation threshold, and resveratrol production), combine genome-wide association studies (GWAS) to map superior alleles, and pyramid them into main cultivars through molecular marker-assisted breeding or gene editing.

In conclusion, the field of physiological and biochemical research on grapevine downy mildew resistance has entered a new stage from phenomenological description to mechanistic dissection and integrated application. Multidisciplinary integration (plant physiology, molecular biology, bioinformatics, and microbiomics) will provide a solid foundation for ultimately achieving green and sustainable management of downy mildew.

## Figures and Tables

**Table 1:** Physiological responses of grapevines to *P. viticola* infection.

Indicator	Change After Infection	Resistance Implication	References
Stomatal traits	Variable	Affect pathogen entry; interplay between stomatal immunity and pathogen-induced opening	[9,10,11,12,13,14,15]
Chlorophyll content	Decrease	Lower reduction in resistant cultivars	[25,26]
Fv/Fm	Decrease	Early disease indicator; reflects PSII damage	[27,28,30,31,32]
Net photosynthetic rate (*P_n_*)	Decrease	Photosynthetic inhibition	[26,32,34]
Transpiration rate (*T_r_*)	Decrease	Gas exchange limitation	[26,32]
Intercellular CO_2_ concentration (*C_i_*)	Increase	Non-stomatal limitation (mesophyll cell damage)	[26,32,34]
Soluble sugars	Dynamic (depends on infection stage)	Early accumulation as signals/energy; later fluctuations related to source-sink balance	[26,36,37,38,39,40,41,42,43,44,45,46,47]
Malondialdehyde (MDA)	Increase	Membrane lipid peroxidation; indicates membrane damage	[18,19,20,21,22,23,24]

**Table 2:** Biochemical defense responses associated with grapevine resistance.

Component	Function	Response in Resistant Cultivars	Response in Susceptible Cultivars	References
SOD	Scavenges superoxide anions (O_2_^−^)	Rapid early induction; converts O_2_^−^ to H_2_O_2_ signal	Delayed response or passive stress-induced increase	[33,48,49,50,51,52,53,54]
POD	Lignification, cell wall reinforcement, phenolic oxidation	Strong activation; changes in isozyme profile	Total activity may increase but functional differentiation insufficient	[19,48,55,56,57,58,59,60]
CAT	Decomposes high-concentration H_2_O_2_	Maintained or increased at late stage; prevents oxidative damage	Activity often decreased or was inactivated due to release	[45,48,61,62]
APX	Fine regulation of H_2_O_2_ (high affinity)	High activity; efficient AsA-GSH cycle regeneration	Lower activity; weak regeneration system	[63,64,65,66,67]
PAL	Rate-limiting enzyme of phenylpropanoid pathway	Rapid, sustained, and strong induction	Weak or delayed induction	[21,69,70,71,72,73,74,75]
PPO	Oxidizes phenolics to quinones (antimicrobial, cross-linking)	Increased activity; effective activation of proenzyme	Lower activity or protein degradation	[76,77,78,79,80]
CHT (Chitinase)	Pathogenesis-related protein; degrades pathogen cell walls	Strong induction; synergistic with GLU	Weak induction	[81,82,83,84,85,86,87,88]
GLU (β-1,3-glucanase)	Pathogenesis-related protein; degrades oomycete cell walls	Strong induction; secreted isoforms crucial	Weak induction	[81,82,83,84,85,86,87,88]
Resveratrol (Stilbene)	Phytoalexin; inhibits pathogen	Rapid accumulation; efficient STS gene expression	Lower accumulation	[89,90,91,92,93,94,95]

## Data Availability

The original contributions presented in this study are included in the article. Further inquiries can be directed to the corresponding authors.

## Abbreviations

The following abbreviations are used in this manuscript:

*P. viticola*	*Plasmopara viticola*
POD	Peroxidase
SOD	Superoxide dismutase
SA	Salicylic acid
JA	Jasmonic acid
MAPK	Mitogen-activated protein kinase
PR	Pathogenesis-related
PAMPs	Pathogen-associated molecular patterns
ROS	Reactive oxygen species
MDA	Malondialdehyde
PSII	Photosystem II
Fv/Fm	Photochemical efficiency
NADPH	Nicotinamide adenine dinucleotide phosphate (reduced form)
ATP	Adenosine triphosphate
NPQ	Non-photochemical quenching
*P_n_*	Net photosynthetic rate
*T_r_*	Transpiration rate
*C_i_*	Intercellular CO_2_ concentration
CAT	Catalase
APX	Ascorbate peroxidase
PRX	Peroxidases
AsA	Ascorbate
MDHAR	Monodehydroascorbate reductase
DHAR	Dehydroascorbate reductase
GR	Glutathione reductase
PAL	Phenylalanine ammonia-lyase
PPO	Polyphenol oxidase
CHT	Chitinase
GLU	*β*-1,3-glucanase
DAMPs	Damage-associated molecular patterns
STS	Stilbene synthase
PKS	Polyketide synthase
PTI	PAMP-triggered immunity
RBOH	Respiratory burst oxidase homolog
CDPKs	Calcium-dependent protein kinases
ET	Ethylene
SAR	Systemic acquired resistance
